# Neurobehavioral, biochemical and histological assessment of the effects of resveratrol on cuprizone-induced demyelination in mice: role of autophagy modulation

**DOI:** 10.1007/s13105-023-00959-z

**Published:** 2023-05-03

**Authors:** Doaa M. Samy, Eiman I. Zaki, Passainte S. Hassaan, Doaa A. Abdelmonsif, Dalia Y. Mohamed, Samar R. Saleh

**Affiliations:** 1 Department of Medical Physiology, Faculty of Medicine, Alexandria University, Alexandria, Egypt; 2 Department of Histology and Cell Biology, Faculty of Medicine, Alexandria University, Alexandria, Egypt; 3 Medical Biochemistry Department, Faculty of Medicine, Alexandria University, Alexandria, Egypt; 4 Center of Excellence for Research in Regenerative Medicine and Applications (CERRMA), Faculty of Medicine, Alexandria University, Alexandria, Egypt; 5 Department of Clinical Pharmacology, Faculty of Medicine, Alexandria University, Alexandria, Egypt; 6 Department of Biochemistry, Faculty of Science, Alexandria University, Alexandria, Egypt; 7 Bioscreening and Preclinical Trial Lab, Faculty of Science, Alexandria University, Alexandria, Egypt

**Keywords:** FoxO1, LC3-II/LC3-I, MBP, Multiple sclerosis, SIRT1, SQSTM1/p62

## Abstract

Resveratrol is known to exhibit neuroprotective effects in many neurological disorders via autophagy modulation. However, controversial results have been reported about the therapeutic potential of resveratrol and the implication of autophagy in demyelinating diseases. This study aimed to evaluate the autophagic changes in cuprizone-intoxicated C57Bl/6 mice and explore the effect of autophagy activation by resveratrol on the demyelination and remyelination processes. Mice were fed with chow containing 0.2% cuprizone for 5 weeks, followed by a cuprizone-free diet for 2 weeks. Resveratrol (250 mg/kg/day) and/or chloroquine (an autophagy inhibitor; 10 mg/kg/day) were given for 5 weeks starting from the third week. At the end of the experiment, animals were tested on rotarod and then sacrificed for biochemical assessment, luxol fast blue (LFB) staining, and transmission electron microscopy (TEM) imaging of the corpus callosum. We observed that cuprizone-induced demyelination was associated with impaired degradation of autophagic cargo, induction of apoptosis, and manifest neurobehavioral disturbances. Oral treatment with resveratrol promoted motor coordination and improved remyelination with regular compacted myelin in most axons without a significant impact on myelin basic protein (MBP) mRNA expression. These effects are mediated, at least in part, via activating autophagic pathways that may involve SIRT1/FoxO1 activation. This study verified that resveratrol dampens cuprizone-induced demyelination, and partially enhances myelin repair through modulation of the autophagic flux, since interruption of the autophagic machinery by chloroquine reversed the therapeutic potential of resveratrol.

## Introduction

Demyelinating diseases, such as multiple sclerosis (MS), are a group of pathological conditions characterized by demyelination, inflammation, and neurodegeneration. Available medications have a limited ability to stop or reverse neurodegeneration and neurological deficit in demyelinating diseases. However, targeting myelin repair “remyelination” may be the most promising treatment approach to reserve neuronal dysfunction and prevent axonal loss, which may improve clinical prognosis in these diseases.

Autophagy is the process of intracellular digestion of misfolded or aged proteins, intracellular pathogens, and damaged organelles, including mitochondria, endoplasmic reticulum, and peroxisomes [16]. It is believed that impairment of autophagy and toxic accumulation of these products may threaten cell survival and end in apoptosis [41]. In fact, the implication of autophagy in the pathogenesis of MS is not clearly understood. It is still controversial whether autophagy leads to cell death in MS or acts as a part of the endogenous neuroprotective responses, since autophagy plays an opposing role in cell survival and apoptosis [21].

Cuprizone (Bis(cyclohexanone) oxaldihydrazone) animal model is a widely used model to investigate the processes of demyelination and remyelination in the central nervous system (CNS). Cuprizone is considered to induce a well-defined and highly reproducible pattern of nonimmune-mediated demyelination [25]. The underlying mechanism of demyelination is still not fully understood. However, it is suggested that cuprizone preferentially affects mature oligodendrocytes and inhibits the copper-dependent mitochondrial enzymes, which in turn disturbs energy metabolism and leads to oxidative stress and induction of apoptosis. These mechanisms ultimately result in the disrupted synthesis of myelin lipid and protein resulting in myelin disintegration [27]. Previous studies showed that five weeks intake of 0.2% cuprizone-enriched chow induces acute demyelination of the corpus callosum. However, natural endogenous remyelination occurs during subsequent weeks when mice are reverted to a normal diet [40].

The natural phytoalexin, resveratrol (3, 5, 4'-trans-trihydroxystilbene) is a polyphenolic compound, found in red grapes, blueberries, and many plant species. Resveratrol is well known for its pleiotropic neuroprotective effects against many neurodegenerative diseases through its anti-inflammatory, antioxidant, and antiaging properties [14]. Activation of the silent information regulator 1 (SIRT1) is believed to play a key role in the neuroprotection conferred by resveratrol in brain tissues [26]. SIRT1 is a NAD-dependent deacetylase involved in mammalian cell DNA repair, senescence, proliferation, and energy metabolism [31]. Recent studies showed that SIRT1 alleviates neurotoxicity by activating autophagy [2, 6]. Additionally, SIRT1 could directly promote the expression of the autophagy machinery components via their deacetylation or indirectly deacetylate transcription factors, which in turn activate several autophagy genes. Amongst these are the Forkhead Box subgroup-O (FoxO) family members [6, 17].

Resveratrol was reported to provide neuroprotection and prevent neurotoxicity via enhancing autophagy [42]. Yet, its exact impact on the autophagic machinery in MS has not been evaluated. Therefore, in the present study, we investigated the autophagic changes in cuprizone-induced demyelination in the mouse model and shed light on the possible influence of autophagy modulation by resveratrol on this nonimmune model of MS. Furthermore, we verified the effect of interruption of the autophagic machinery by chloroquine on the therapeutic potential of resveratrol.

## Material and Methods

### Animals

Male 8–9-week-old C57BL/6 mice, weighing 25–30 g (procured from the Experimental Animal Centre of Alexandria University, Alexandria, Egypt) were used. Mice were acclimatized to standard laboratory conditions including 12 h light/dark cycle, temperature 22 ± 1 °C and free access to standard powdered chow and tap water for one week before starting the experiment.

### Chemicals

Cuprizone, chloroquine, and dimethyl sulfoxide (DMSO) were obtained from Sigma-Aldrich Co (Fluka, USA), while resveratrol was purchased from Biotivia Longevity Bioceuticals (USA). Other chemicals were obtained with high grades.

### Demyelination and experimental design

Animals were given 0.2% (w/w) cuprizone mixed with the standard ground rodent chow for 5 weeks for acute induction of demyelination. The standard ground rodent chow containing cuprizone was changed every other day. After week 5, animals fed with cuprizone-mixed chow were given a cuprizone-free chow for an additional 2 weeks to allow for spontaneous remyelination [40]. Two weeks following the start of the cuprizone, animals were randomly divided into four experimental groups (*n* = 14 mice/group): cuprizone-intoxicated (CPZ) group, resveratrol-treated group (CPZ + RSV), chloroquine-treated (CPZ + CHQ) group, and resveratrol and chloroquine-treated (CPZ + RSV + CHQ) group. The resveratrol-treated groups received 250 mg resveratrol/kg/day by oral gavage [14] dissolved in physiological saline containing 1% DMSO, whereas the groups treated with chloroquine received *i.p.* injection of 10 mg chloroquine/kg/day, dissolved in PBS [18]. All drugs were prepared immediately before administration.

**Fig. 1:**
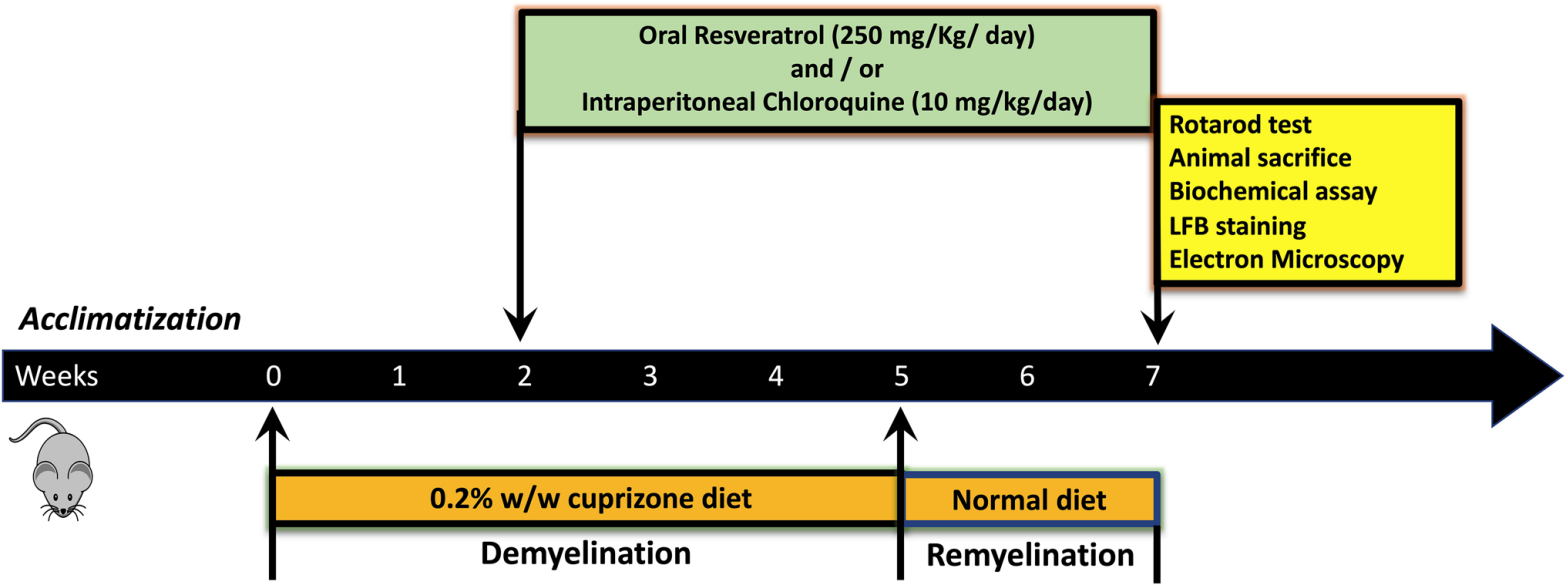
Experimental design of the study. Mice were fed with chow containing 0.2% cuprizone for 5 weeks to induce demyelination, followed by a cuprizone-free diet for additional 2 weeks. Starting from the third week, animals received oral resveratrol and/or chloroquine, or vehicle-treated for 5 weeks. Afterward, motor coordination was assessed on rotarod, and animals were then sacrificed for biochemical analysis and electron microscopic imaging of the corpus callosum

Additional three experimental control groups (*n* = 14 mice/group) were fed standard rodent chow without cuprizone throughout experimental weeks and received 1% DMSO in saline *p.o.* and PBS *i.p.* as vehicles (Control group), resveratrol (RSV group) or chloroquine (CHQ group) at the same doses mentioned above. Figure 1 shows an illustration of the schedule for induction of demyelination/remyelination and drug administration.

### Neurobehavioral testing:* rotarod test.*

After 7 weeks, all animals were subjected to neurobehavioral assessment. The rotarod test was used to evaluate balance and locomotor coordination in mice. On the training day, mice were placed on the rotarod for 5 min at 16, then 24, and finally 32 rpm. On the second day, mice were placed on the rotarod for 5 min at 16, then 24 rpm, and allowed to rest for 1 h. Then, animals were tested at 32 rpm for 5 min. The time each mouse was able to stay on the rotarod before the first fall (latency time) was recorded, and the number of falls was counted [39]. A fall was counted every time the animal fell completely off the rotating rod. If a mouse fell during the first 10 s of a trial, it was replaced on the rotating rod.

### Tissue* c*ollection and preparation

At the end of the experimental period, mice were euthanized by decapitation under anesthesia, and brain tissues were dissected and washed in ice-cold phosphate buffer saline. For histochemical examination of the myelin, brains of 3 mice/group were fixed in 10% buffered formol saline, and then 5 μm coronal sections were stained with luxol fast blue (LFB).

For the rest of the animals, corpus callosum tissues were isolated under a dissecting microscope, using a scalpel blade, from coronal cuts at about Bregma − 0.25 mm and − 1.25 mm. Then, sagittal cuts were done bilaterally through the cingulum, medial to the lateral ventricle. The cortex and fornix were gently removed from above and below the corpus callosum, respectively [35].

Corpus callosum samples were immediately processed for either transmission electron microscopy (TEM) examination (*n* = 3/group) or assessment of biochemical parameters (*n* = 8/group). For the determination of mRNA expression by quantitative reverse transcription PCR (qRT-PCR), one part of the corpus callosum was kept in RNA later solution (Ambion, Austin-TX, USA) and stored at − 80 °C. The other part was snap-frozen in liquid nitrogen and kept at − 80 °C for protein expression analysis by western blot.

### Histochemical staining

For LFB staining, tissue sections were deparaffinized with xylol and hydrated with decreasing alcohol concentrations. Afterward, the tissue was incubated in 0.1% LFB solution overnight at 60 °C. The next day, the excess stain was rinsed off with 95 and 70% ethyl alcohol (each for 3 min), rinsed in distilled water, and differentiated in 0.05% lithium carbonate solution. Subsequently, counterstaining with hematoxylin for 1 min was performed. The tissue was then washed and dehydrated with increasing alcohol concentrations. Tissue sections were examined under a light microscope, and morphometric analysis of the percentage of the myelinated area was performed using the NIH Fiji program (NIH, Bethesda, MD, USA).

### Quantitative RT-PCR

Total RNA extraction from corpus callosum samples was performed following the manufacturer’s instructions of RNeasy Mini Kit (Qiagen, GmBH-Germany) and reverse-transcription was done using SuperScrip™ III Reverse Transcriptase (RT) kit (Thermo Fisher Scientific, USA). Quantitative RT-PCR was then performed in 25 μl reaction volume using 1X SYBR® Green PCR Master Mix (Thermo Fisher Scientific, USA) in StepOne real-time PCR system (Thermo Fisher Scientific, USA). The used primers (obtained from Invitrogen, Thermo Fisher Scientific, USA) are shown in Table 1. The protocol for amplification included one cycle at 95 °C for 2 min with subsequent 40 cycles of denaturation at 95 °C for 5 s, an annealing step as shown in Table 1, and an extension step at 72 °C for 20 s. Amplification was followed by melting curve analysis and negative control was included with each PCR run. The comparative CT method was used for the calculation of the expression of the targeted genes via StepOne™ Software v2. relative to the housekeeping gene glyceraldehyde 3 phosphate dehydrogenase (GAPDH).

**Table 1:** Primer sequences used in qRT-PCR

Name/size/ accession No	Forward	Reverse	Tm (ºC)	Ref
GAPDH/308/ NM_017008.4	TCCCTCAAGATTGTCAGCAA	AGATCCACAACGGATACATT	52	[29]
MBP/103/ NM_017026.2	CCCATTGGTGCACACTAACCT	CGACTTGATTCAGCGACAGGA	65	[38]
LC3-II/138/ NM_022818.5	GAGAAGCAGCTTCCTGTTCTGG	GTGTCCGTTCACCAACAGGAAG	60	[19]
SQSTM1/P62/145/ NM_003900.5	TGCCCAGACTACGACTTGTG	AGTGTCCGTGTTTCACCTTCC	60	[36]

*GAPDH*, glyceraldehyde 3-phosphate dehydrogenase; *MBP*, myelin basic protein; *LC3-II*, microtubule-associated protein light chain-3 II; *SQSTM1/P62*, sequestosome 1/polyubiquitin-binding protein p62

### Western Blot Analysis

Total protein extraction from corpus callosum samples was achieved using RIPA buffer [150 mM sodium chloride, 1% Triton × 100, 0.5% sodium deoxycholate, 1% sodium dodecyl sulphate (SDS), and 50 mM Tris (pH 8)] containing protease inhibitor cocktail (Sigma-Aldrich, USA), 1 mM phenylmethylsulfonyl fluoride and 1 mM sodium orthovanadate. Lysate was next incubated on ice for 20 min and centrifuged (10,000 g, 15 min, 4 °C). The Bio-Rad protein assay kit protocol (Bio-Rad, Mississauga, Canada) was adopted for the determination of lysate protein concentration. Subsequently, 30 μg of each sample protein was denatured by boiling for 5 min and then subjected to SDS–polyacrylamide gel electrophoresis (SDS-PAGE). Bio-Rad Trans-Blot apparatus was utilized for protein blotting to nitrocellulose membranes (Bio-Rad, Mississauga-Canada). Membrane blocking was done using 5% bovine serum albumin (for 1 h at room temperature) and membranes were incubated with the specific primary antibodies (overnight at 4 °C). Membrane wash in 1X Tris-buffered saline-tween 20 (TBST; pH 7.4) was performed with subsequent incubation with the proper secondary antibody (for 2 h at room temperature). Membrane wash was then accomplished in TBST and protein bands were visualized using 3, 3', 5, 5'-tetramethylbenzidine stain. Antibodies dilutions were as follows: LC3 A/B (#4108S, Cell Signaling Technology, 1:1000), SQSTM1/p62 (#5114, Cell Signaling Technology, 1:1000), SIRT1 (#ab104833, Abcam, 1: 1000), Forkhead Box protein O1 (FoxO1, #2880, Cell Signaling, 1:1000), acetylated-FoxO1 (Ac-FoxO1, #sc49437, Santa Cruz Biotechnology, 1:1000), cleaved caspase 3 (#9664, Cell Signaling Technology, 1:1000) and β-actin (#A5316, Sigma-Aldrich, 1:4000). Protein bands were quantified via Quantity One software (Bio-Rad Laboratories, USA). The levels of protein expression were normalized to that of β-actin and their relative expression was calculated as a fold change of control.

### Transmission electron microscopy and Morphometric Analysis

Corpus callosum samples were cut immediately after dissection into small pieces (0.5–1 mm^2^) and fixed in a 3% glutaraldehyde solution. Ultrathin sections were prepared for TEM examination. Digital images obtained using TEM (JEOL JEM-2100F; Tokyo, Japan) were used to measure the g-ratio. The g-ratio, calculated by dividing the diameter of the axon by the total fiber diameter (axon plus myelin sheath), is a measure of myelin thickness and the remyelination response [7]. Since spontaneously remyelinated fibers are characterized by abnormally thin myelin compared to normal myelinated axons, an increased g-ratio indicates spontaneous remyelination [9]. Unmyelinated axons were not included in g-ratio determinations. In addition, digital images were used to determine the percentage of myelinated axons in each group. Measurements were expressed using NIH Image J (v1.49) software.

### Statistical analysis

Data were described as means ± standard deviation (S.D.). The normality of data distribution was assessed using Shapiro–Wilk’s test. Significant differences between values were analyzed by one-way ANOVA followed by post hoc Tukey test. For the rotarod test, significant outliers were identified by the ROUT method (coefficient *Q* = 1.0%) and excluded from the analysis. Statistical significance was set at *p* < 0.05. The Statistical Package for Social Sciences 20.0 for Windows (SPSS, Chicago, IL) was used for calculation.

## Results

### Effect of resveratrol on cuprizone-induced neurobehavioral disturbance

**Fig. 2:**
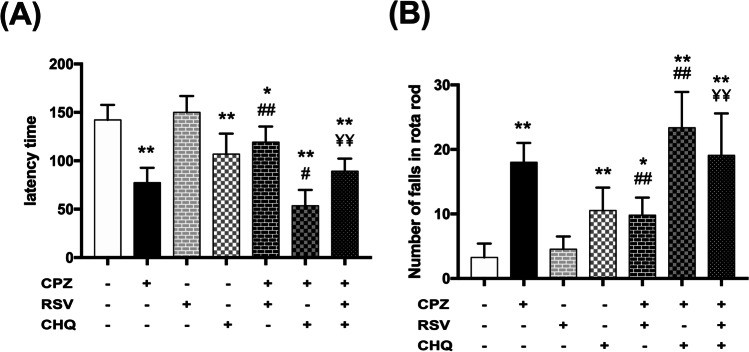
Effect of resveratrol on cuprizone-induced neurobehavioral disturbance. Rotarod test was used. Mice were tested on the rod at 32 rpm for 5 min. The time each mouse was able to stay on the rotarod before first fall (latency time) was recorded (**A**), and the number of falls (**B**) was counted. If a mouse fell during the first 10 s of the test, it was replaced on the rotating rod. The data are presented as the means ± S.D; ^*^p < 0.05, ^**^p ≤ 0.001 compared with control; ^#^*p* < 0.05, ^##*p*^ ≤ 0.001 compared with CPZ group; ¥¥ *p* ≤ 0.001 compared with CPZ + RSV group

Cuprizone-intoxicated mice developed impaired coordination and locomotion manifested by a significant decrease in latency time and a significant increase in the number of falls in the rotarod test compared to the control mice (Fig. 2). Resveratrol treatment in cuprizone-intoxicated mice corrected this disturbed neurobehavior by significantly increasing the latency time and decreasing the number of falls. However, this improvement was significantly different from the control mice. To test whether autophagy plays a role in resveratrol-mediated improvement, resveratrol was combined with the late autophagy inhibitor, chloroquine, for the treatment of cuprizone-intoxicated animals. We observed that chloroquine significantly abolished the protective effects of resveratrol by increasing the number of falls and decreasing the latency time. These results coincided with the cuprizone-intoxicated group treated with chloroquine alone, showing a deteriorated performance in the rotarod test (Fig. 2).

### Effect of resveratrol on cuprizone-induced demyelination/remyelination (assessed by LFB staining, TEM, and myelin basic protein mRNA expression)

**Fig. 3:**
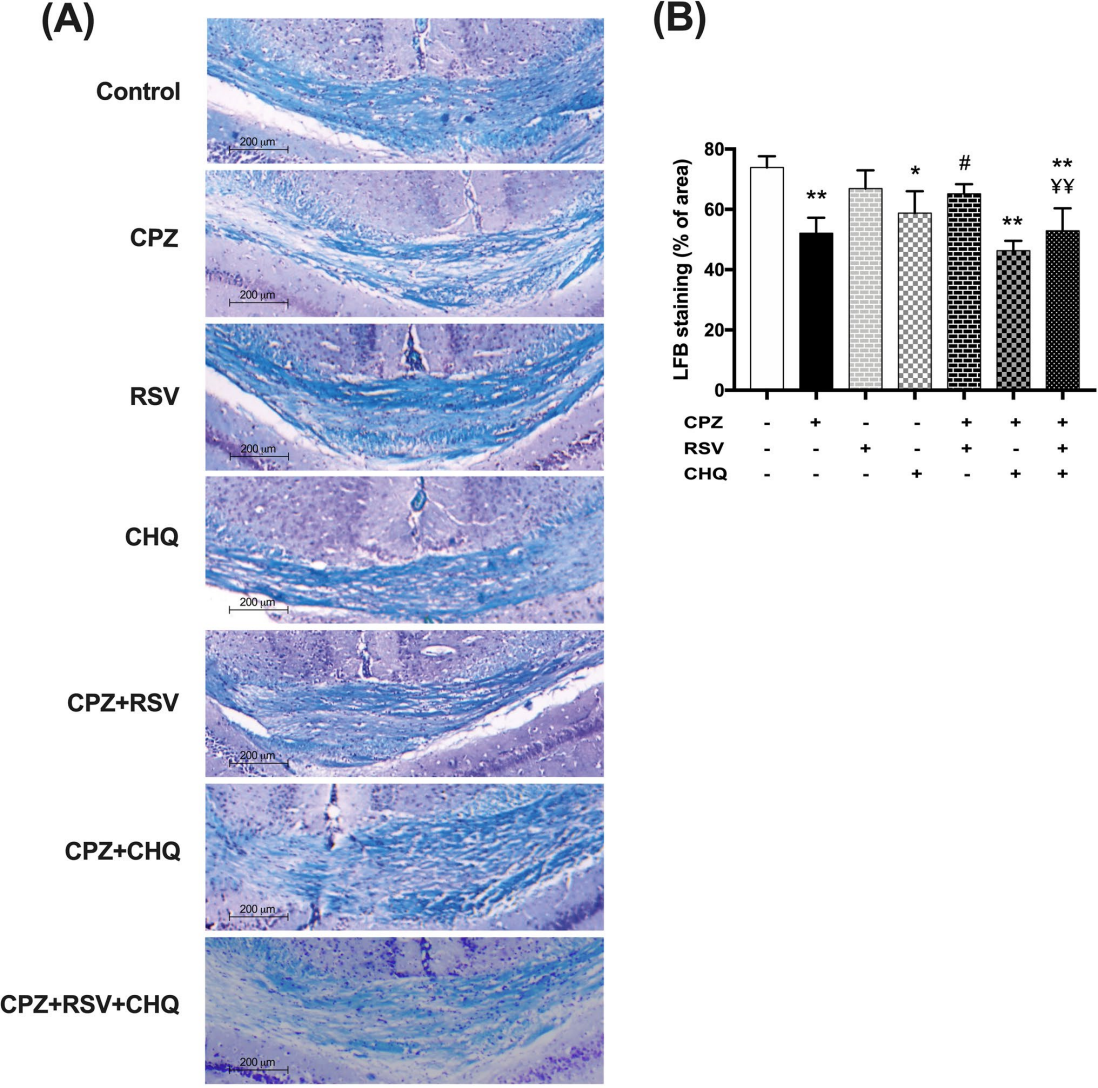
Effect of resveratrol on cuprizone-induced myelin loss in LFB staining. **A** Representative micrographs of the corpus callosum stained with luxol fast blue (LFB). **B** Morphometric analysis of the myelinated area (%). The data are presented as the means ± S.D.; ^*^p < 0.05, ^**^p ≤ 0.001 compared with control; ^#^*p* < 0.05, ^##*p*^ ≤ 0.001 compared with CPZ group; ¥¥ *p* ≤ 0.001 compared with CPZ + RSV group

Histochemical staining of the myelin with LFB revealed a decrease in myelinated areas in the corpus callosum of cuprizone-intoxicated mice, compared to control mice, whereas resveratrol treatment of cuprizone-fed mice significantly increased the myelinated areas in the corpus callosum. Worthy of note, chloroquine cotreatment with resveratrol impaired the remyelination response and decreased the LFB-stained myelinated areas in the corpus callosum in comparison to the CPZ + RSV group (Fig. 3).

**Fig. 4:**
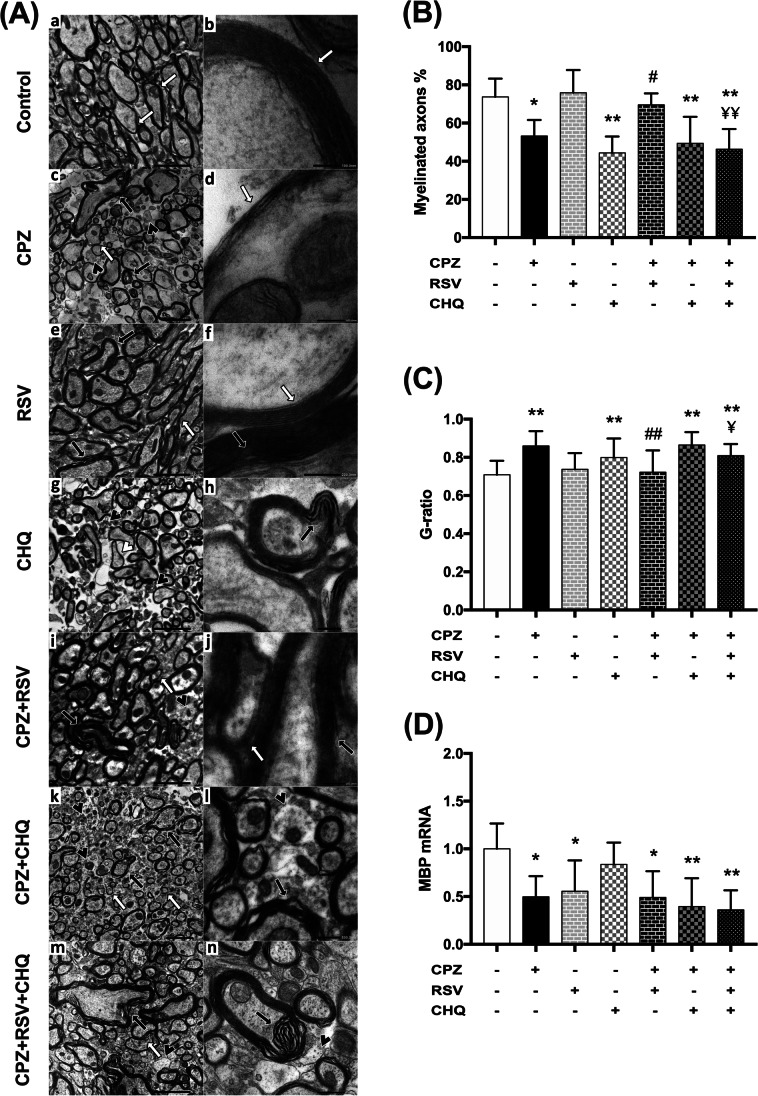
Effect of resveratrol on cuprizone-induced changes in myelin ultrastructure and expression of MBP mRNA. **A** Representative electron microscopy images of the corpus callosum by using uranyl acetate/lead citrate stain. (a, b) Control group showing, myelinated nerve fibers with normal thickness and regularly arranged lamellae of the myelin sheath (white arrow). (c, d) CPZ group showing, unmyelinated nerve fibers (black arrowhead), and myelinated nerve fibers with decreased thickness (white arrow) and disturbed arrangement of the lamellae of the myelin sheath (black arrow). (e, f) RSV group showing, myelinated nerve fibers, with normal thickness and regularly arranged lamellae of the myelin (white arrow), but some excessively myelinated axons with separation in myelin configuration are also seen (black arrow). (g, h) CHQ group showing, unmyelinated nerve fibers (black arrowhead), and myelinated nerve fibers with decreased thickness (white arrowhead) and disturbed arrangement of the lamellae of the myelin sheath (black arrow). (i, j) CPZ + RSV group showing some unmyelinated axons (black arrowhead), and some myelinated axons with normal thickness and regularly arranged lamellae (white arrow) and others are excessively myelinated with separation in myelin configuration (black arrow). (k, l) CPZ + CHQ group showing poorly myelinated (white arrow), unmyelinated nerve fibers (black arrowhead), and myelinated nerve fibers with disturbed arranged lamellae of the myelin (black arrow). (m, n) CPZ + RSV + CHQ group showing, unmyelinated axons (black arrowhead) and some myelinated axons with disturbed lamellae arrangement (white arrow), while others are excessively myelinated with separation in myelin configuration (black arrow). Microscopic Magnifications: a = X4000, b = X60K, c = X3000, d = X60K, e = X4000, f = X40K, g = X4000, h = X25K, i = X4000, j = 25 K, k = X3000, l = X12K, m = X4000, n = X12K. **B** Percentage of myelinated axons as demonstrated by transmission electron microscopy (TEM) imaging of the corpus callosum. **C** The g-ratio, a measure of myelin thickness and the remyelination response, was calculated by dividing the diameter of the axon by the total fiber diameter (axon plus myelin sheath). **D** mRNA expression of MBP in the corpus callosum. Expression levels were normalized to the housekeeping gene glyceraldehyde 3-phosphate dehydrogenase (GAPDH) and expressed as folds of control. The data are presented as the means ± S.D. ^*^p < 0.05, ^**^p ≤ 0.001 compared with control; ^#^*p* < 0. 05, ^##^*p* ≤ 0.001 compared with CPZ group; ¥ *p* < 0.05, ¥¥ *p* ≤ 0.001 compared with CPZ + RSV group

Furthermore, demyelination and remyelination responses were examined on the ultrastructural level, using TEM imaging of the corpus callosum and morphometric analysis. Two weeks following cuprizone cessation, we observed persistent profound demyelination in the CPZ group confirmed by a significant reduction in the percentage of myelinated axons relative to the control group (Fig. 4A, B, and C). This was associated with a significantly higher g-ratio, consistent with the thin and disturbed arrangement of the lamellae of the myelin sheaths, which is characteristic of the spontaneous remyelination response after cuprizone withdrawal. Treatment with resveratrol to diseased animals (CPZ + RSV group) significantly increased the number of myelinated axons and lowered the g-ratio indicating increased myelin thickness and enhanced remyelination with compacted, regularly arranged myelin in most axons. However, some myelinated axons showed poor compaction and separation in myelin configuration. This dysregulated myelin was also observed in some axons of the RSV group, but the g-ratio and the percentage of myelinated axons are comparable to those of the control group. Co-administration of chloroquine with resveratrol for treatment of cuprizone toxicity (CPZ + RSV + CHQ group) deteriorated the improvement in myelin condition conferred by resveratrol, as a higher g-ratio and a lower number of myelinated axons were revealed. Furthermore, administration of chloroquine to cuprizone-fed mice (CPZ + CHQ group) significantly induced demyelination and poor remyelination with disturbed myelin sheath lamellae (Fig. 4A, B, and C).

On the molecular level, an assessment of myelin basic protein (MBP) mRNA expression in the corpus callosum revealed that cuprizone markedly reduced MBP mRNA expression as compared to control mice. Resveratrol monotherapy (CPZ + RSV group) or combined with chloroquine (CPZ + RSV + CHQ group), failed to improve MBP mRNA expression relative to the CPZ group. Interestingly, MBP mRNA expression was significantly downregulated in the RSV group and non-significantly lower in the CHQ group relative to the control group (Fig. 4D).

### Effect of resveratrol on* cuprizone*-induced* autophagic* changes

**Fig. 5:**
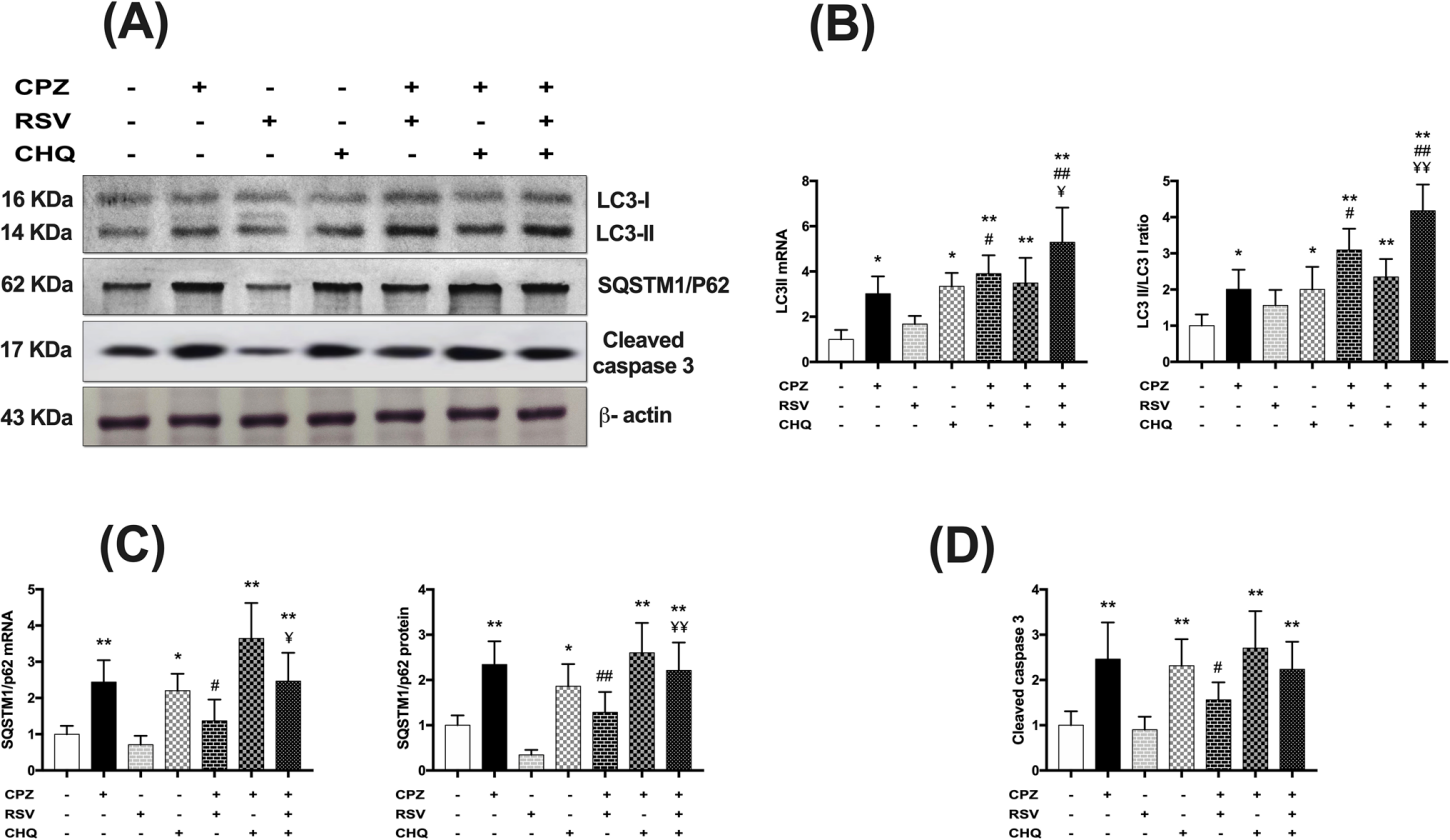
Effect of resveratrol on cuprizone-induced autophagic changes and apoptosis. **A** Representative immunoblots for autophagy and apoptosis-related proteins. **B** LC3II mRNA expression and the ratio of LC3-II/LC3-I protein expression, indicating the conversion of the soluble form (LC3-I) in the cytosol to the autophagosome-bound form (LC3-II). **C** mRNA and protein expression levels of SQSTM1/p62. **D** Protein expression of cleaved caspase 3. The levels of protein expression were normalized to that of β-actin and their expression was calculated relative to control, while the mRNA expression levels were normalized to the housekeeping gene (GAPDH) and expressed as fold change. The data are presented as the means ± S.D; ^*^p < 0.05, ^**^p ≤ 0.001 compared with control; ^#^*p* < 0. 05, ^##^*p* ≤ 0.001 compared with CPZ group; ¥*p* < 0.05, ¥¥*p* ≤ 0.001 compared with CPZ + RSV group

Autophagic machinery was evaluated via assessment of LC3-I, LC3-II, and p62 accumulation in the corpus callosum. In this study, increased LC3-II mRNA expression and higher LC3-II/LC3-I protein ratio were observed in the CPZ group in comparison with the control group. Treatment with resveratrol in the CPZ + RSV group further increased LC3-II mRNA expression as well as the LC3-II/LC3-I protein ratio as compared to the CPZ group (Fig. 5A and B).

In fact, the conversion of the soluble form (LC3-I) in the cytosol to the autophagosome-bound form (LC3-II) and consequently increased LC3-II/LC3-I ratio indicates either an increase in autophagosome formation, which is typically proportional to increased autophagic flux, or instead denotes autophagosome accumulation due to inhibition of autophagic flux at lysosomal level [24]. To differentiate between these two potentials, we measured the protein and mRNA expression of another autophagic marker, SQSTM1/p62. Increased p62 is indicative of protein build-up due to the inhibition of lysosomal fusion to the autophagosome. Therefore, p62 accumulates when autophagy is inhibited and decreases when autophagy is activated [5]. As shown in Fig. 5C, cuprizone administration significantly increased p62 protein and mRNA expression levels, whereas resveratrol alone decreased them. Notably, p62 was significantly lower in the CPZ + RSV group than in the CPZ group, suggesting that cuprizone suppresses autophagic degradation, while resveratrol induces lysosomal degradation to complete the autophagic flux.

Chloroquine is known to block the late stage of autophagolysosomal formation, and thus, as expected, it increased the LC3-II/LC3-I ratio. Interestingly, the highest ratios were observed in the CPZ + RSV + CHQ group (Fig. 5A and B). Moreover, chloroquine reversed the effect of resveratrol on p62 protein and mRNA expression levels in the CPZ + RSV + CHQ group, whereas it did not affect the cuprizone-induced elevation of p62 expression levels when it was given to the CPZ group (Fig. 5C).

Collectively, these results imply that cuprizone hindered the autophagic machinery downstream of autophagosome formation by inhibiting autophagic degradation, while resveratrol promoted autophagosome formation and facilitated autophagic protein degradation.

### Effect of resveratrol on cuprizone-induced apoptosis

The cleaved caspase-3 expression level was assessed in different experimental groups to determine whether treatment with resveratrol possesses an anti-apoptotic effect in the corpus callosum. A significant increase in cleaved caspase-3 was found in cuprizone-intoxicated mice as compared to the control group. Resveratrol treatment reduced cleaved caspase-3 expression in the corpus callosum, whereas chloroquine co-treatment with resveratrol abolished this anti-apoptotic effect in the cuprizone-intoxicated mice (Fig. 5D).

### Effect of resveratrol on* cuprizone*-induced SIRT1/FoxO1 pathway inhibition

**Fig. 6:**
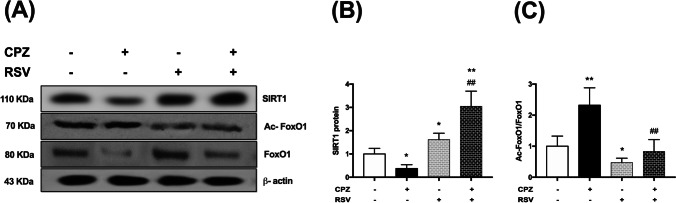
Effect of resveratrol on cuprizone-induced SIRT1/FoxO1 pathway inhibition. **A** Western blot analysis for SIRT1, Ac-FoxO1, and FoxO1 proteins in the corpus callosum. **B** Protein expression of SIRT1. **C** Ratio of Ac-FoxO1/FoxO1 protein expression levels. The levels of protein expression were normalized to that of β-actin and their expression was calculated relative to control. The data are presented as the means ± S.D; ^*^p < 0.05, ^**^p ≤ 0.001 compared with control; ^##^*p* ≤ 0.001 compared with CPZ group

As shown in Fig. 6, cuprizone intoxication caused a marked decline in corpus callosum SIRT1 protein expression. On the other hand, oral resveratrol treatment (CPZ + RSV group) significantly increased the expression of SIRT1. As FoxO1 is a substrate for SIRT1 in the autophagic cascade, we evaluated the protein levels of FoxO1 and Ac-FoxO1. Ac-FoxO1 expression was significantly increased by cuprizone intoxication and significantly decreased following resveratrol treatment (Fig. 6). These data suggest that resveratrol enhances the deacetylation of FoxO1 by SIRT1 overexpression. It is worth mentioning that resveratrol administration in cuprizone-free mice also caused a significant upregulation of the SIRT1/FoxO1 signaling pathway.

### Discussion

Multiple sclerosis, the most common demyelinating disease, results in devastating long-term neurological dysfunction, partly due to a lack of effective remyelination in the adult human brain. Therefore, identification of new therapeutic targets to enhance remyelination is a key goal in MS treatment. The complexity of the pathophysiologic mechanisms of MS makes none of its experimental animal models exactly mimic human pathology. However, the cuprizone-induced animal model of MS has an advantage in evaluating the demyelination/remyelination process as compared to other models of MS [25]. In cuprizone-intoxicated mice, we observed persistent extensive demyelination and poor defective remyelination response after two weeks of cuprizone withdrawal. This was associated with blocked degradation of autophagic cargo, induction of apoptosis in the corpus callosum, and manifest neurobehavioral disturbances.

Studies investigated the role of naturally derived polyphenol, and resveratrol, in different models of demyelinating diseases and reported ambiguous results. In our study, resveratrol significantly improved balance and coordination in the rotarod test, alleviated apoptosis, decreased the number of demyelinated axons, and increased the thickness of regenerated myelin in the corpus callosum. However, not all the repaired myelin was compacted and regularly arranged. Furthermore, resveratrol failed to ameliorate the downregulated MBP. In line with our results, Ghaiad et al*.* [15] showed a complex neuroprotective effect for resveratrol against the cuprizone model of MS, but MBP in their study was upregulated by resveratrol. Other reports have revealed that resveratrol attenuates neuronal damage and prevents neuronal loss in relapsing–remitting and chronic experimental autoimmune encephalomyelitis (EAE) models of MS [12, 32]. In contrast to these promising results, resveratrol treatment exacerbated demyelination and inflammation without neuroprotection in both viral and EAE models [30]. Also, a recent study revealed that the resveratrol application to the MK-801-induced mouse model of schizophrenia does not correct behavioral disorders or demyelination and even reduces MBP expression [1]. It is believed that these conflicting effects of resveratrol may be due to differences in the severity and course of diseases, the different strains of animals used, or protocols of therapy.

MBP, a major myelin protein, plays an integral role in the process of myelin arrangement and compaction by lipid clustering [14]. In the present study, cuprizone induced a significant reduction in MBP mRNA expression causing very thin defective myelin repair two weeks after cuprizone withdrawal, as confirmed by our histological findings. Indeed, MBP deficiency disrupts myelin compaction and stability, leading to myelin degradation. A puzzling finding in this study was that oral administration of resveratrol had no effect on MBP mRNA expression in cuprizone-intoxicated mice and even decreased MBP in normal mice. This may explain the presence of some dysregulated uncompacted myelin in both groups, though most myelinated axons had normal multilamellar compacted myelin. Indeed, the myelin formation is a complex process, in which many other myelin-related proteins play a pivotal role in providing structure and integrity to the myelin sheath, such as myelin proteolipid protein (PLP), 2,3-cyclic nucleotide 3- phosphodiesterase (CNPase) and myelin oligodendrocyte glycoprotein (MOG), which merit further investigation. Remarkably, the observable difference in motor performance in the rotarod test between resveratrol-treated animals and control animals, and the incomplete improvement of balance and coordination after resveratrol treatment as compared to normal may highlight that restoration of MBP is necessary to fully rescue the motor function.

Accumulating evidence suggests that autophagy plays a crucial role in the pathogenesis of demyelination and remyelination processes in the CNS [5]. In our model, cuprizone toxicity increased both LC3-II/LC3-I ratio and SQSTM1/p62 expression, denoting impairment of autophagy progression and interruption of autophagic flux downstream to autophagosome formation. These results are comparable with previous findings in acute and chronic EAE, where the accumulation of protein aggresomes in the spinal cord and reduced LC3-II/LC3-I ratio were correlated with impaired autophagic protein turnover mechanisms [8]. Also, a recent study attributed cuprizone-induced demyelination to autophagy inhibition [2]. Oppositely, a study on the effect of cuprizone on the corpus striatum myelin in a schizophrenia model reported that autophagy is increased by cuprizone administration [10]. The reason for this contradiction may be explained by a difference in the experimental designs. Indeed, the time course alterations in autophagic response to different doses of cuprizone need further verification.

Although the association between autophagy and pathogenesis of demyelination is poorly understood, it is possible that aggregation of myelin debris may dysregulate cellular trafficking and impair lysosomal function and autophagy accomplishment. In turn, defective clearance of myelin may contribute to the impairment of the myelination process [33]. Furthermore, induction of autophagy may be a unique cellular mechanism that promotes the survival and function of oligodendrocytes, while suppression of the autophagic pathway may trigger apoptosis of oligodendrocytes [3, 33]. In accordance with previous reports, cuprizone increased the expression of the apoptotic marker caspase-3, which was suppressed by resveratrol treatment. A recent study demonstrated that maturation and differentiation of oligodendrocyte progenitor cells to myelinating oligodendrocytes, the sole providers of myelin in the CNS, are tightly regulated by autophagy, where the genetic or pharmacological inhibition of autophagy blocks myelination [3]. Meanwhile, the administration of rapamycin, a potent autophagy inducer, ameliorates the EAE model of MS [11] and improves myelination and neuronal survival in tuberous sclerosis [23]. Similarly, autophagy enhancement in Long-Evans Shaker rats, which is characterized by a mutation in MBP and severe CNS demyelination, increases the number of myelinated axons and myelin sheath thickness [33]. On the contrary, other studies have shown that autophagy directly participates in the progression of EAE [4].

Recently, induction of autophagy was suggested as a direct therapeutic target for myelin repair in demyelinating diseases [2, 3], and resveratrol proved effective in reversing some neurological disorders via autophagy activation [42]. Therefore, it was interesting to investigate the implication of autophagy in resveratrol neuroprotective effects in cuprizone-induced demyelination. Our results revealed that resveratrol restored the cuprizone-suppressed autophagic activity and enhanced lysosomal degradation, as revealed by high LC3-II/LC3-I ratio and low SQSTM1/p62 expression, in addition to increased autophagosomes and autolysosomes in TEM images.

Chloroquine is a membrane-permeant lysosomal inhibitor that inhibits autophagosome-lysosome fusion by altering the acidic environment of lysosomes, thereby preventing the final degradation of autophagic cargo [22]. In our work, blocking autophagic degradation using chloroquine abolished the protective effects of resveratrol on the cuprizone model of MS in the combined-treated mice, and exacerbated functional and histological abnormalities in cuprizone-intoxicated mice. These findings propose that autophagy induction may be a key mediator of resveratrol beneficial responses in this demyelination model. Interestingly, injection of chloroquine to control mice resulted in some deterioration in motor functions as well as axonal demyelination and irregular myelin configuration; validating the postulation that autophagy is a crucial process for normal myelination.

One of the principal target proteins for resveratrol action is SIRT1. SIRT1 regulates several biological processes related to axonal integrity, cell differentiation, mitochondrial metabolism, apoptosis as well as autophagy [20, 42]. Therefore, it is the most investigated member of the sirtuin family in different animal models of MS. Several autophagic pathways are activated by SIRT1, including deacetylation of prominent transcription factors such as the FoxO family that promotes the expression of several autophagy genes. It was previously reported that SIRT1 increases deacetylation, activation, and nuclear translocation of FoxO1 [17], which in turn increases the expression of Rab7, a small GTPase that facilitates autophagosome-lysosome fusion; hence it plays an essential role in the late phase of the autophagic process [37].

Our results revealed that SIRT1 and FoxO1 were suppressed in the corpus callosum by cuprizone toxicity, while resveratrol successfully reversed this decrease in SIRT1 and FoxO1 deacetylation. Therefore, in our study, we believe that autophagy was interrupted at a late stage in cuprizone-intoxicated mice, and the induction of autophagic flux and the achievement of autophagic degradation by resveratrol may be mediated by SIRT1/FoxO1 pathway activation. However, due to the complex correlation between MS and autophagy, the other underlying mechanisms and the associated signaling pathways involved in resveratrol-induced autophagy activation in cuprizone-intoxicated animals need to be more elucidated.

The role of SIRT1 in MS in experimental research is a matter of debate; numerous investigations reported that SIRT1 activation can ameliorate the course of the disease and improve neurological functions by targeting several neuroprotective pathways [32], whereas, in other studies, remyelination and neuroprotection were seen following SIRT1 inhibition [28]. This discrepancy in results may be explained by the differences in the pathogenicity of MS models and the duration of experiments. Indeed, alteration of this protein in lesion sites of MS patients implies a potential role of SIRT1 modulation in demyelinating diseases that needs more clarification [34].

**Fig. 7:**
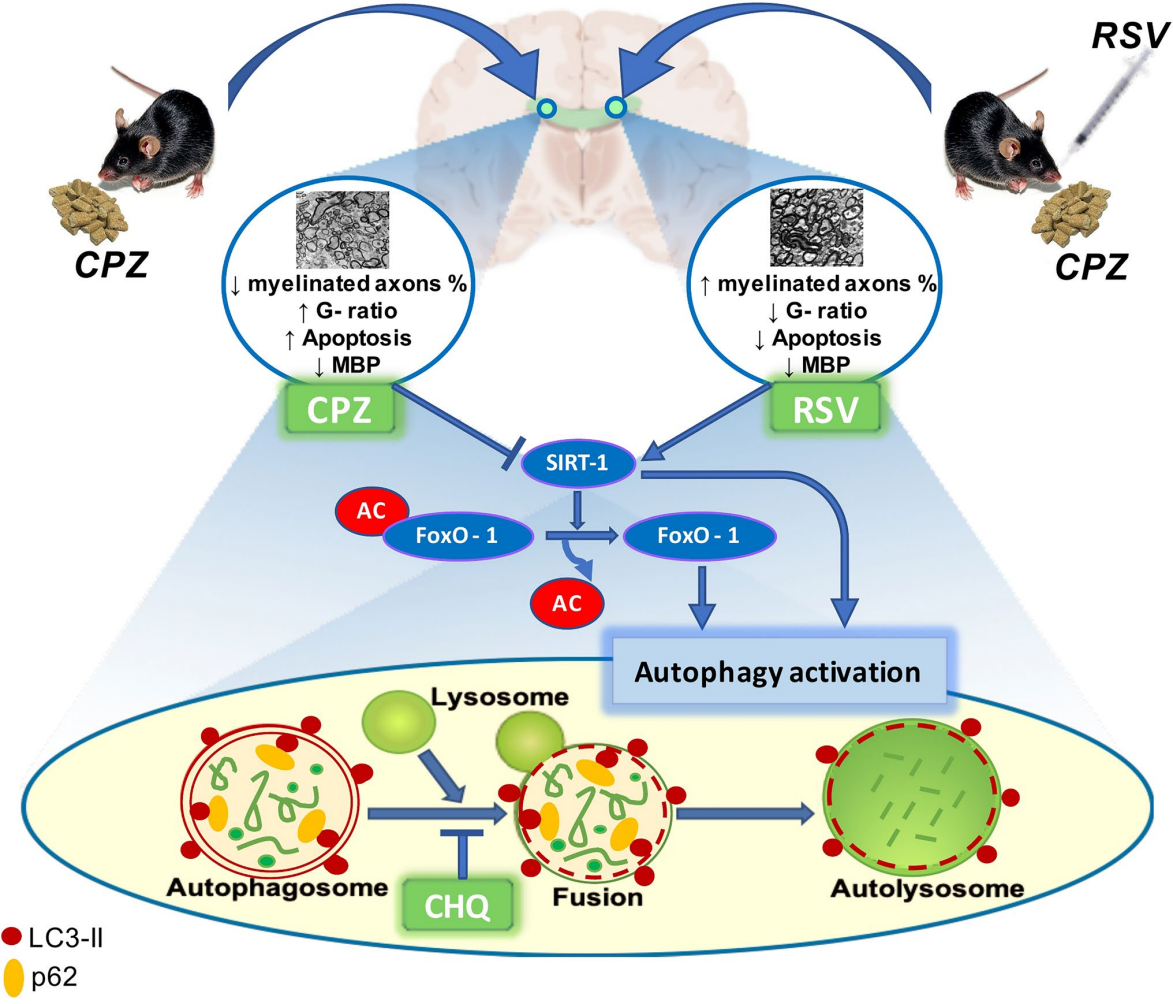
Resveratrol improves remyelination in cuprizone mouse model of demyelination probably by SIRT1/FOxO1 pathway-mediated autophagy

In conclusion, our results suggest that the autophagic machinery is dysregulated in the cuprizone model of MS and highlight a partial therapeutic potential of resveratrol against MS by improving motor deficits, suppressing apoptosis, alleviating demyelination, and enhancing remyelination. However, some regenerated myelin layers were uncompacted and irregular, probably due to persistent downregulation of MBP. In addition, we proposed, for the first time, that the benefits of resveratrol could be mediated by the activation of autophagic pathways, which may involve SIRT1/FOxO1 pathway activation (Fig. 7). One limitation of this study is that oligodendrocyte survival or apoptotic changes after cuprizone and/or resveratrol treatment were not evaluated by immunostaining or high magnification TEM. More experiments need to be carried out in order to show that resveratrol has a beneficial effect on the MS-affected nervous system through autophagic activity modulation. However, this work may represent a small step in this direction.

## Data Availability

The data of this study are available from the corresponding author upon reasonable request.
